# Hybridized triboelectric nanogenerators for simultaneously scavenging droplet and wind energies

**DOI:** 10.3389/fchem.2024.1538660

**Published:** 2025-01-10

**Authors:** Chaosheng Hu, Chengmin Bao, Yang Liu, Yingzhan Yan, Yanan Bai, Qian Xu

**Affiliations:** ^1^ Information Science Academy, China Electronics Technology Group Corporation, Beijing, China; ^2^ School of Chemistry and Environmental Engineering, Hohhot Minzu College, Hohhot, China; ^3^ National Key Laboratory of Integrated Circuits and Microsystems, Beijing, China; ^4^ School of Physical Science and Technology, Guangxi University, Nanning, China

**Keywords:** wind, droplet, triboelectric nanogenerator, hybridized, energy

## Abstract

Triboelectric nanogenerators (TENGs) have attracted great attention due to the simple manufacturing process, low cost, and diverse forms of energy harvesting. However, the energy collected by individual TENG is relatively limited, making it necessary to develop new method to enhance the energy harvesting capability of TENG. Here, we design a hybridized TENG that integrates a droplet-driven TENG and a wind-driven TENG, which exhibits excellent electrical performance. Under the simulated environment of medium rain with medium breeze, the hybridized TENG generates an output voltage of 95.10 V, a maximum average power of 18.15 μW, an energy of 181.54 μJ in 10 s, and charges a 1 μF capacitor to 43.29 V in 120 s. This work enables the harvesting of dispersed wind and droplet energy from the environment, providing new ideas and possibilities for online monitoring in remote areas and the construction of Internet of Things systems.

## 1 Introduction

With the rapid advancements in technology, the demand for energy has shown an unprecedented growth trend. This trend has also prompted the development of various new forms of energy, including solar energy (Zhang et al., 2021), thermal energy (Kumar and Mandal, 2024), mechanical energy (Qu et al., 2023), wind energy (Wang et al., 2021), ocean energy (Xu et al., 2023), and nuclear energy (2023). With the booming development of IoT technology, IoT devices have become ubiquitous, ranging from smart homes to smart cities (Madavarapu et al., 2024; Kim et al., 2021) and from industrial monitoring to environmental sensing (Garcia et al., 2022; Filho et al., 2021). All these applications rely on a stable and continuous energy supply. Therefore, efficiently and conveniently harvesting clean energy from the environment has become a critical issue for researchers to address. Based on this, energy harvesting units based on triboelectric nanogenerators (TENGs) have stood out due to their exceptional energy harvesting capabilities (He et al., 2021; He et al., 2023a; He et al., 2023b; Pang et al., 2021). TENGs not only feature simple manufacturing processes and low costs but also exhibit extremely diverse forms of energy harvesting, making them flexible and adaptable to various application scenarios. These characteristics have rapidly positioned TENGs as a highly notable research direction. TENGs are capable of capturing energies that are often overlooked in daily life. Whether it is slight vibrations of objects (Wang L. et al., 2024), the sliding motion between objects (Tang et al., 2022), human respiration (Zhang et al., 2019), the fluctuation of waves (Wang Y. et al., 2024), or the falling of raindrops (Xu et al., 2020), all of these seemingly negligible energies can be effectively collected by TENGs. After appropriate conversion and storage, the collected energy can support the normal operation of self-powered electronic devices (Gao et al., 2024; Kim et al., 2024; Su et al., 2021), providing a solid energy guarantee for the long-term stable operation of IoT devices.

The wind-driven triboelectric nanogenerators (W-TENGs) have been extensively reported due to their simple structure and ease of installation, encompassing various designs such as rotating (Li et al., 2024), single-ended fixed (Son et al., 2023), and double-ended fixed (Chen et al., 2018) configurations. W-TENGs are typically installed in open areas like deserts, grasslands, and rooftops to maximize wind energy harvesting. In addition, the droplet-driven TENG (D-TENG) has been increasingly favored as a new energy harvesting method in recent years (Lee et al., 2024; Zhang et al., 2022; Dong et al., 2022), which harvests the energy generated by falling water droplets during rainy days, which is also easy to be ignored. Both of these generators can effectively harvest small amounts of energy from the environment, but certain limitations exist. First, they are commonly suitable for relatively simple environments during the energy harvesting process and face challenges in adapting to complex and changing environments. Second, the amount of energy collected is not sufficient to power electronic components.

In this paper, we have designed a hybrid nanogenerator that integrates D-TENG and W-TENG by optimizing the device structure. Compared to traditional W-TENG, the droplet and wind-driven hybrid generator (DW-TENG) achieves the simultaneous harvesting of wind and water droplet energy with almost no increase in volume. By comparing the output performance of D-TENG and W-TENG when operating independently and simultaneously, it is evident that the output power and energy of the hybrid generator are significantly superior to those when the generators are operating alone, indicating that the hybrid generator can harvest more energy. Additionally, by investigating the impact of parameters such as wind speed and droplet frequency on the performance of the hybrid generator, we have obtained the optimal parameters for achieving the best performance, providing a valuable reference for the external environmental conditions during actual energy harvesting processes. Furthermore, to demonstrate the practical application value of the hybrid generator, it was installed on the top of a yurt. Under conditions where both wind and rain occur simultaneously, the device still exhibited good output performance. Additionally, the capacitor charging curve and the ability to light up LEDs also demonstrated its excellent energy output capability, laying a solid foundation for the research on self-powered electronic devices. This work fully exploits the advantage of TENGs in simultaneously harvesting different forms of energy, especially under windy and rainy conditions, providing a good reference for the research on efficient energy harvesting and self-powered electronic devices in complex natural environments.

## 2 Experimental section

### 2.1 Manufacturing of D-TENG

A piece of acrylic sheet cut using a laser cutting machine (PLS4.75, General laser Systems, United States) was used as the lower support layer. A piece of Al foil used as the bottom electrode with the same size as the acrylic sheet was pasted onto the acrylic sheet. Then, a layer of friction film with the same size as the acrylic sheet and the Al foil was glued on the Al electrode. Finally, a piece of elongated Al foil was glued to the fluorinated ethylene propylene (FEP) film as the top electrode. Different types of friction films (thickness, 30 μm), such as FEP, polytetrafluoroethylene (PTFE), Kapton, polyvinyl chloride (PVC), waterproof cloth, and nylon, were used according to the experimental conditions. The different sizes of devices (30 mm × 30 mm, 40 mm × 40 mm, 50 mm × 50 mm, 100 mm × 50 mm, and 75 mm × 75 mm), different lengths (10 mm, 15 mm, 20 mm, 25 mm, 30 mm, 40 mm, and 50 mm), and widths (0.5 mm, 1 mm, 2 mm, 3 mm, 4 mm, and 5 mm) of the Al foil (the top electrode) were used to optimize the output performance, respectively. The device was glued to acrylic panels with varying angles of inclination.

### 2.2 Manufacturing of the W-TENG

The W-TENG prepared in this paper is a double-ended fixed wind-driven structure TENG, which consists of a friction film as the friction layer and two Al electrodes. Two acrylic plates (100 mm × 10 mm × 3 mm), cut using a laser cutting machine (PLS4.75, General laser Systems, United States), were used as the upper and lower support layers, with 5-mm diameter holes (two) at both ends. The Al foil electrode was adhered to the acrylic board. A gap was left between the friction film and the Al foil electrode to ensure sufficient friction between them, and a 5-mm hole was reserved at the corresponding position on both the friction film and the Al foil for fixing. Then, by installing bolts and screws on both sides of the fixing device, the double-ended fixed wind-driven TENG was assembled. Different types of friction films (thickness, 30 μm), such as FEP, PTFE, Kapton, PVC, waterproof cloth, and nylon, were used according to the experimental conditions. Shims of varying thicknesses (1 mm, 1.5 mm, 2 mm, 2.5 mm, and 3 mm) were used to adjust the gap between the friction film and the aluminum foil electrode. The device was glued to acrylic panels with varying angles of inclination.

### 2.3 Manufacturing of the hybridized DW-TENG

The hybridized DW-TENG consists of a D-TENG and a W-TENG, as manufactured above, with the W-TENG on the lower level and D-TENG on the upper level. The D-TENG and W-TENG share the middle Al electrode; the bottom electrode of the D-TENG is used as the top electrode of the W-TENG. Two acrylic plates (100 mm × 50 mm × 3 mm), cut using a laser cutting machine (PLS4.75, General laser Systems, United States), were used as the upper and lower support layers, with 5-mm-diameter holes (four) at four corners. Two pieces of the Al foil electrode of 100 mm × 10 mm were adhered to each acrylic board. A 2.5-mm gap was left between the FEP friction film and the Al foil electrode, and a 5-mm hole was reserved at the corresponding position on the FEP film and the Al foil for fixing. The FEP friction film can be fixed on the long side of the rectangle acrylic plates. The upper Al foil electrode was adhered to the lower surface without covering the entire surface and was then attached to cover the whole upper surface of the acrylic plate, extending from the side edges of the acrylic plate in succession. This Al foil served as both the top electrode of the W-TENG and the bottom electrode of the D-TENG. Then, another piece of the FEP film of 100 mm × 50 mm was glued on the top of the upper layer of the Al foil electrode. At last, a piece of 50 mm × 2 mm elongated Al foil was adhered to the FEP film. In addition, the whole hybridized device was glued to an acrylic panel with a 45° angle of inclination.

### 2.4 Characterization and measurements

**FIGURE 1:**
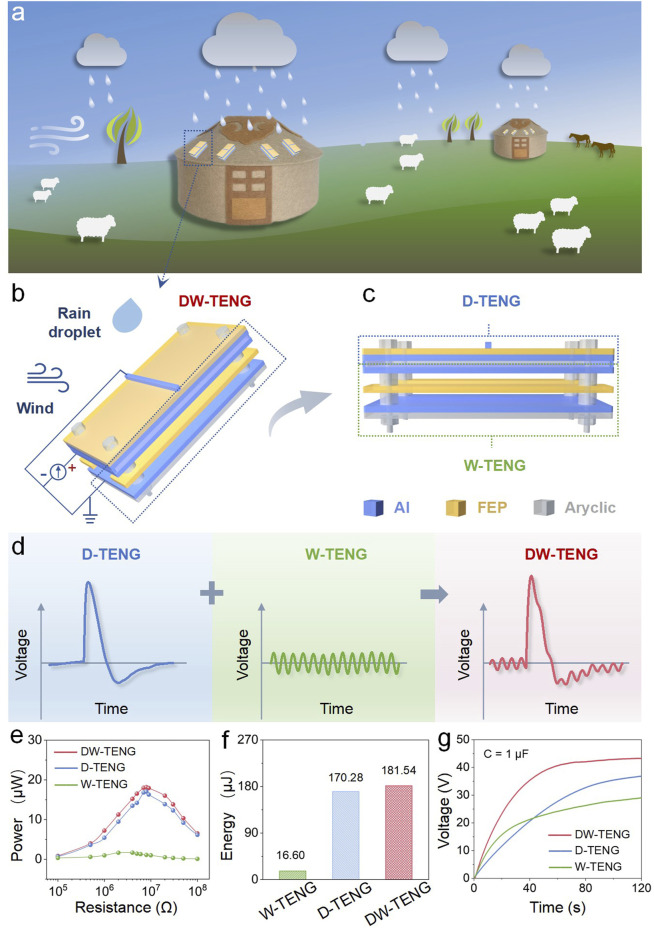
Schematic diagram and output performance of the DW-TENG. **(A)** Demonstration diagram of DW-TENG application scenarios. **(B, C)** Schematic diagram of the structure of the DW-TENG. **(D)** Voltage signal graphs of the W-TENG, D-TENG, and DW-TENG. **(E)** Power output comparison, **(F)** energy generation comparison, and **(G)** speed comparison of 1-μF capacitor charge. Operation conditions: droplet frequency = 10 Hz, wind speed = 5 m/s, dripping height = 30 cm, and angle = 45°.

The output voltage and current of TENGs were tested using a Keithley 6514 (Keithley) electrometer. The output signal of the DW-TENG in Figures 1E–G, 4A–D was measured using a single side link and a single-FEP film swing. A beaker filled with water was placed on an iron stand that could be adjusted in height. Water was pumped from the beaker using a syringe pump and plastic tubing. A 36-cm-diameter cylinder fan with 140 W power was used to generate wind (300, BANJEKT). The wind speed is measured using a handheld anemometer (DLX-ANE2302, DELIXI ELECTRIC).

## 3 Results and discussion

### 3.1 Structure and output performance of the DW-TENG

In order to scavenge both energies simultaneously, we assembled droplet- and wind-driven TENGs, called DW-TENG, that can harvest both droplet and wind energy and convert them to electric energy. This electric energy converted from dispersed energy in the environment can be used to detect environmental temperature, humidity, or wind speed in remote areas such as yurts, where online monitoring is not popular, as shown in Figure 1A. The device consists of a W-TENG at the bottom and a D-TENG at the top. The W-TENG consists of upper and lower Al electrodes and a friction layer (FEP) in the middle, with a gap between Al and FEP to increase effective friction. The upper D-TENG consists of a bottom Al electrode, a friction layer of FEP, and an upper elongated Al foil from bottom to top. W-TENG and D-TENG share the middle aluminum electrode, which means that the top electrode of the W-TENG and the bottom electrode of the D-TENG are the same piece of aluminum. When working, the shared Al electrode in the middle serves as the positive electrode, and the top Al electrode of the D-TENG and the bottom Al electrode of the W-TENG are connected in parallel as the negative electrode for electric energy output, as shown in Figures 1B, C **.** During operation, the water droplets are positioned above the strip electrodes so that the droplets are just in contact with the strip electrodes when they are completely dispersed on the device surface, which produces the best current (Bao et al., 2023). If the position is too far off, the signal will be reduced, and previous studies have reported the results. The volume per droplet is 60 μL, which is obtained by measuring the total volume of 100 drops of water using a measuring cylinder and dividing by the number of droplets. The thickness of the Al electrode used during the experiment is 20 μm. When the water drops fall from a height of several tens of centimeters, the droplets have a large downward sliding fractional velocity on the surface of the device, and each drop of water will wash away the residual droplets from the previous drop, so it is not easy to gather easily. As shown in Figure 1D, D-TENG outputs a pulse-like voltage signal and W-TENG outputs periodic voltage signals with smaller amplitude but higher frequency; when the two are hybridized in parallel, the voltage outputs are superimposed on each other. To further investigate the relationship between DW-TENG, D-TENG, and W-TENG, the output power and energy of DW-TENG were measured and calculated. When a resistor is connected, the voltage of the load can be measured, and the power can be calculated according to Equation 1. By changing the resistance value, the relationship curve between resistance and power can be obtained, thus obtaining the maximum power. In addition, the output energy can be calculated by Equation 2. The Equations 1, 2 are as follows:

$$P=\frac{U^{2}}{R},$$ (1)

$$E=\int_{0}^{t}\frac{U^{2}}{R}dt.$$ (2)

Here, *U* represents the voltage, *R* represents the load resistance, and *t* represent the time.

Figure 1E shows the maximum average power of the D-TENG, W-TENG, and DW-TENG. The maximum average power values were 17.03 μW, 1.66 μW, and 18.15 μW at load resistances of 8 MΩ, 4 MΩ, and 8 MΩ, respectively. In other words, the output energy of the D-TENG, W-TENG, and DW-TENG in 1 min can be up to 170.28 μJ, 16.60 μJ, and 181.54 μJ, respectively, as shown in Figure 1F. The signal of the W-TENG is smaller than that of the D-TENG, which may be because the contact area of the FEP film as a friction material is too small, resulting in less charge generated by friction. Furthermore, the humidity of the environment near the generator increases due to the effect of water droplets, which reduces the signal of the W-TENG. The presence of a water film may reduce direct contact between materials, thereby reducing the effect of frictional electrification. Although the individual output signal of the D-TENG is larger than that of the W-TENG, the output frequency is smaller than that of the W-TENG. So, the charging performance shows that the charging rate of the W-TENG is faster than that of the D-TENG at the initial charging stage, and it is also shown during the charging process of the hybridized device. As a result, the hybridized generator is able to collect more energy. Moreover, the charging capabilities of TENGs were measured and compared, as shown in Figure 1G. A capacitor of 1 μF could be charged up to 43.29 V, 36.87 V, and 29.08 V by the DW-TENG, D-TENG, and W-TENG, respectively, in 120 s. The abovementioned data show that the D-TENG and W-TENG signals were successfully hybridized together, resulting in an increase in power and energy and improved charging performance.

### 3.2 Working principle of the DW-TENG

**FIGURE 2:**
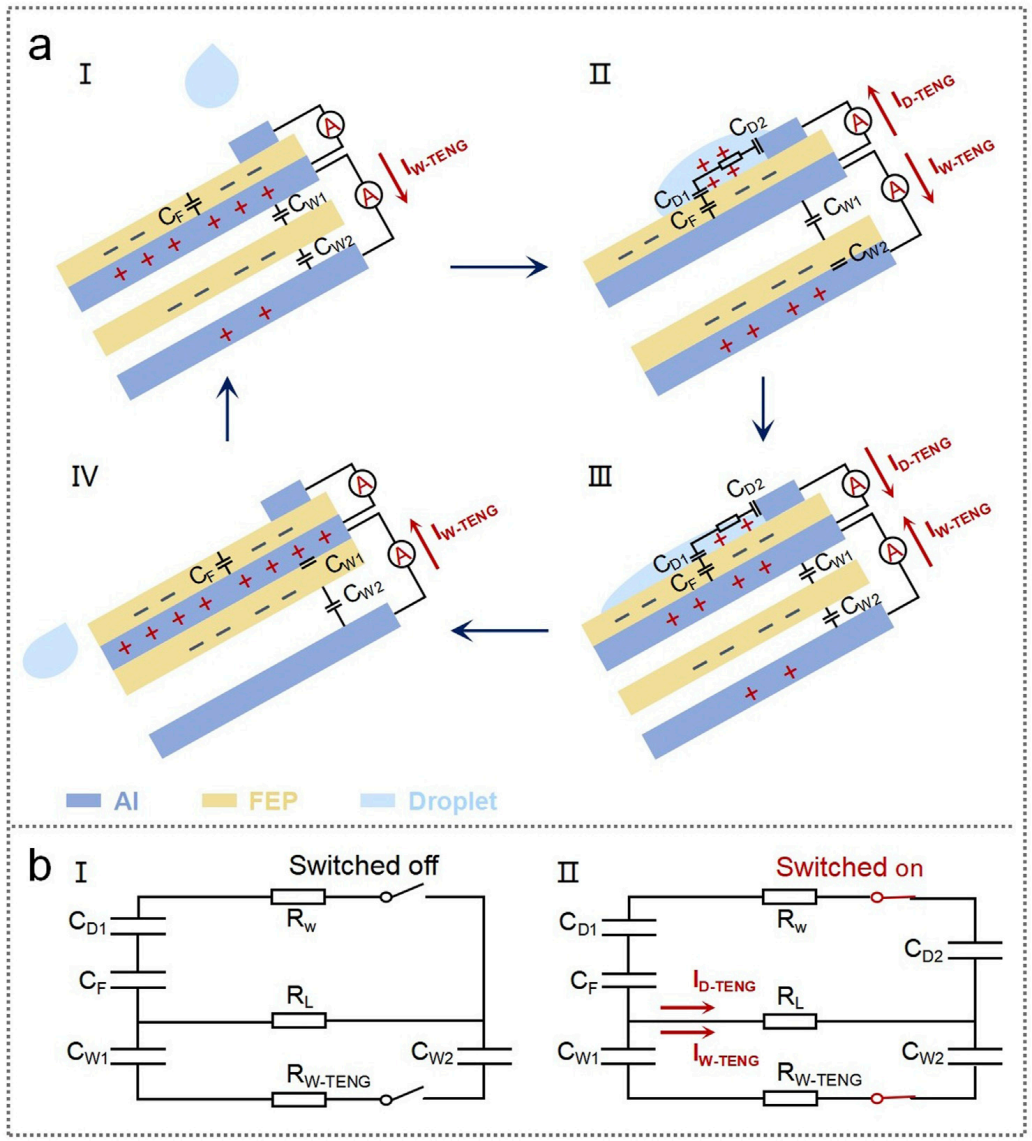
Working principle and equivalent circuit diagram of the DW-TENG. **(A)** Working principle of the DW-TENG: a cycle is divided into four stages (I–IV). **(B)** Equivalent circuit diagram of the DW-TENG.

The working principle is based on electrostatic induction at the liquid–solid and solid–solid interfaces. The working process can be divided into four steps, as shown in Figure 2A. In the first step, when the device is in the normal condition, a certain number of negative charges are accumulated in the FEP layer of the D-TENG and the FEP layer of the W-TENG. An equal number of positive charges are induced both in the Al electrode (the shared electrode) in the middle layer and the Al electrode in the lower layer due to electrostatic induction. At this point, it can be considered that there exists a capacitance C_F_ between the FEP layer of the D-TENG and the middle layer Al. Similarly, capacitances C_W1_ and C_W2_ exist between the FEP layer of the W-TENG and the middle layer Al and the FEP layer of the W-TENG and the lower layer Al, respectively. In the second step, when a water droplet falls on the device and contacts the top Al electrode and the FEP, a capacitance C_D1_ is formed at the interface of the droplet and the FEP. At the same time, another capacitance C_D2_ is formed at the interface of the droplet and the top Al electrode. As a result, C_F_, C_D1_, and C_D2_ constitute a complete circuit. In this case, since the FEP layer originally accumulated a lot of charges, U_F_ > U_D1_, U_D2_ and capacitances C_D1_ and C_D2_ are charged from capacitance C_F_, forming a current flowing from the middle Al electrode to the top Al electrode. Meanwhile, in the portion of the W-TENG, when the FEP swings downward to contact the bottom Al electrode, the induced charges on the middle Al electrode decrease, while the induced charges on the bottom Al electrode increase, forming a current flowing from the middle Al electrode to the bottom Al electrode. In the third step, with the water droplet gradually sliding down the device surface, U_D1_ and U_D2_ gradually increase, and U_F_ gradually decreases. Until U_D1_, U_D2_ > U_F_ and capacitances C_D1_ and C_D2_ recharge to capacitance C_F_ in turn; the direction of the current also changes to flow from the top Al electrode to the middle Al electrode. At the same time, in the portion of the W-TENG, FEP swings upward, resulting in the induced charges decreasing on the bottom Al electrode and the induced charges increasing on the middle Al electrode. Then, the current flowing from the bottom Al electrode to the middle Al electrode is formed. In the fourth step, the water droplet slides off, and the FEP of the W-TENG swings back to the normal condition. To further detail the working principle of the DW-TENG, the equivalent circuit diagram is shown in Figure 2B. When there is no droplet dripping and no wind, the device is in the switched off state, as shown in Figure 2B, and when water is dripping and wind is blowing, it switches on, as shown in Figure 2B. An AC current flowing from the middle Al electrode to the top and bottom Al electrodes and then from the top and bottom Al electrodes back to the middle Al electrode is generated due to electrostatic induction.

### 3.3 Output performance optimization

**FIGURE 3:**
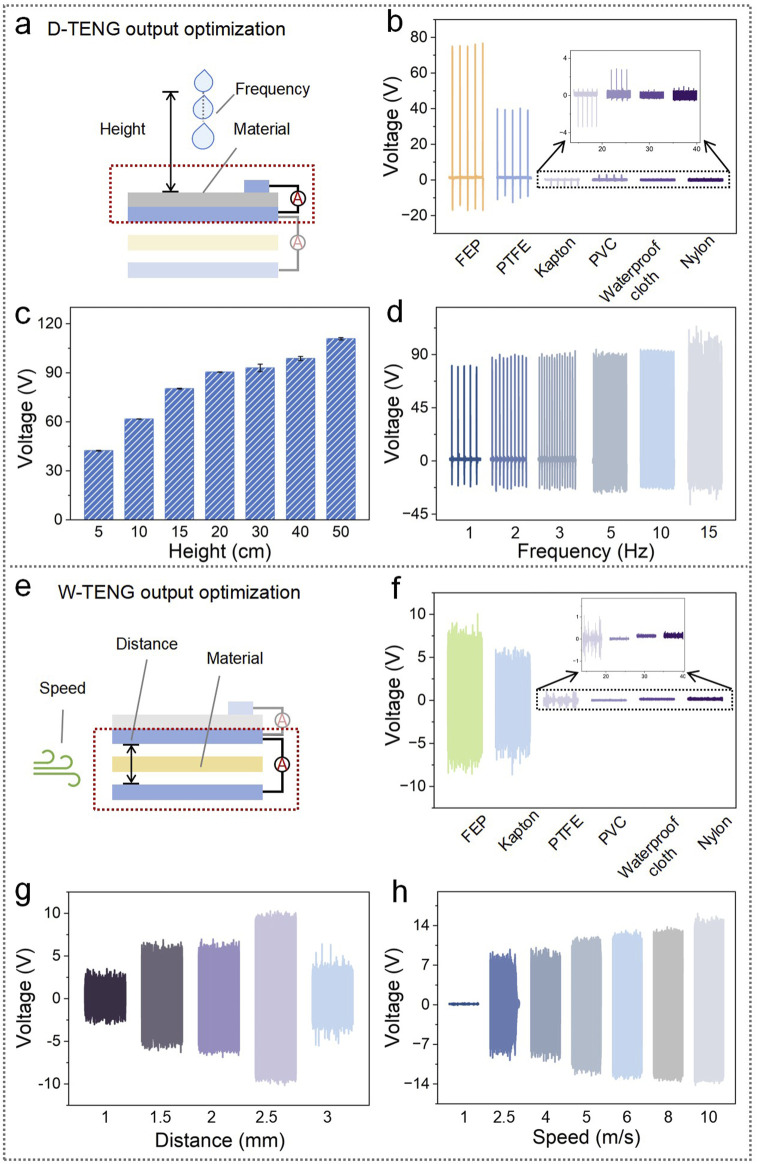
Output optimization for the D-TENG and W-TENG. **(A)** Output optimization content of the D-TENG and **(E)** the W-TENG. **(B)** Output voltage of the D-TENG under different surface materials (size = 30 × 30 mm, frequency = 1 Hz, height = 15 cm, top electrode length = 15 mm, top electrode width = 2 mm, and angle = 45°), **(C)** different droplet heights (size = 100 × 50 mm, top electrode length = 50 mm, top electrode width = 2 mm, frequency = 1 Hz, and angle = 45°), and **(D)** different droplet frequencies (size = 100 × 50 mm, height = 15 cm, top electrode length = 50 mm, top electrode width = 2 mm, and angle = 45°). **(F)** Output voltage of the W-TENG under different friction materials (size = 100 × 10 mm, wind speed = 5 m/s, distance = 1.5 mm, and angle = 0°), **(G)** different gap distances between the FEP and Al electrodes (size = 100 × 10 mm, wind speed = 5 m/s, and angle = 0°), and **(H)** different wind speeds (size = 100 × 10 mm, distance = 2.5 mm, and angle = 0°).

In order to optimize the output performance of the hybridized DW-TENG, each impact factor on the output performance of the D-TENG (Supplementary Figure S1A) and the W-TENG (Supplementary Figure S1B) was investigated separately, as shown in Figures 3A, E. Specifically, of the several different friction materials selected in Figures 3B, F, FEP performs the best for both D-TENG and W-TENG. For the D-TENG, the device with FEP had an average output voltage of up to 75.61 V and an average current of up to 12.49 μA, while the device with PTFE had an average output voltage and current of 39.54 V and 6.78 μA, as shown in Figure 3B and Supplementary Figure S2A. For the W-TENG, the device with the FEP had an average output voltage of up to 7.06 V and an average current of 0.77 μA , while the device with Kapton had an average output voltage and current of 4.15 V and 0.23 μA, respectively, as shown in Figure 3F and Supplementary Figure S5A. FEP was selected as the friction material for the DW-TENG due to its superior performance over other materials. As shown in Figure 3C, the performance of the D-TENG increases significantly with an increase in dripping height. The higher the droplet falls, the greater the Weber number generated (*We* = *ρD*_*0*_ *v* ^*2*^ */γ,* where *ρ* and *D*_*0*_ are the density and diameter of the droplet, respectively, *v* is the impact velocity, and *γ* is the surface tension), and a higher Weber number leads to a larger diffusion area of the droplet (*D*_*m*_ *∼ D*_*0*_ *We* ^*1/4*^, where *D*_*m*_ is the maximum diameter of the droplet), which results in a fuller contact with the FEP and generates more charge. Therefore, the open-circuit voltage gradually increases as the height of the drop increases. In addition, the higher the drop height, the greater the velocity of the droplet when contacting the device. In addition, the partial velocity in the direction of the flat plate will be larger, and thus, greater current will be generated. When FEP was used as the friction layer, the device was inclined at 45°, the droplet frequency was 1 Hz, the droplet dripping height was 50 cm, the average output voltage was up to 110.81 V, and the current was up to 16.96 μA (Supplementary Figure S2B). Figure 3D demonstrates a plot of the D-TENG signal as a function of the droplet frequency, which does not have a significant effect on the magnitude of the output performance. The signal became less stable when the frequency was up to 15 Hz. When the frequency was 10 Hz, the average output voltage could reach 92.86 V, and the current reached 15.77 μA (Supplementary Figure S2C). The optimized data for the inclined angle of the device (Supplementary Figure S3A, B) show that the highest output current and voltage were achieved at an inclined angle of 45°. Moreover, for the W-TENG, Supplementary Figure S6 shows that there are only slight fluctuations in the voltage and current signals at different tilt angles, rather than significant changes in the W-TENG with changes in the angle. Different tilt angles may result in different speeds of droplet sliding on the FEP surface, but this has very little impact on the signal and can be basically ignored, and the device was able to output voltage and current up to 7.84 V and 1.68 μA, respectively, when inclined at 45°. Based on these results, the inclined angle of the hybridized DW-TENG was selected as 45°. In addition, other impact factors such as the size, top electrode length, and top electrode width of the D-TENG were tested, and the results showed that these three factors did not have a significant effect on the output performance of the device (Supplementary Figure S4). The output voltage was in the range of 85–100 V, and the current was in the range of 13–15.5 μA. For the W-TENG, the gap between the FEP and the upper and lower Al electrodes had a more significant influence on the output performance. As shown in Figure 3G and Supplementary Figure S5B, the best output performance was achieved when the gap was 2.5 mm, with an average output voltage of 9.12 V and a current of 1.78 μA. Wind speed was another major output performance influencing factor. The output of the W-TENG increased with wind speed, with an average output voltage and current of 13.04 V and 2.92 μA, respectively, when the wind speed reached 10 m/s, as shown in Figure 3H and Supplementary Figure S5C. In addition, the performance of the W-TENG is related to the size of the device. It has been reported that the best output performance is achieved when the length to width ratio of the W-TENG is 10:1 (Wang et al., 2015), and a related study also carried out experiments with excellent output performance at this size ratio (Zhu et al., 2022). Therefore, the length-to-width values of W-TENGs in this study were determined using the aforementioned ratios. Based on the performance optimization data mentioned above, the size of the hybridized DW-TENG device was selected as 100 × 50 mm, i.e., the size of the FEP layer and the bottom Al electrode of the D-TENG portion was 100 × 50 mm, the length of the top electrode was set at 50 mm, and the width was set at 2 mm. The size of the FEP layer and the top and bottom Al electrodes of the W-TENG was set at 100 mm × 10 mm, the gap between the FEP and Al electrodes was set at 2.5 mm, and the inclined angle was chosen as 45°. The top Al electrode of the W-TENG and the bottom Al electrode of the D-TENG should be a connected sheet of Al foil affixed to both sides of the acrylic support plate. The Al located on the upper surface served as the bottom electrode of the D-TENG with an area of 100 × 50 mm, and the Al attached to the lower surface of the acrylic plate from the side edges of the acrylic plate in succession served as the top electrode of the W-TENG, which had an area of 100 mm × 10 mm in a relatively narrow strip and did not cover the entire lower surface of the acrylic plate.

### 3.4 Output and application of the hybridized DW-TENG

**FIGURE 4:**
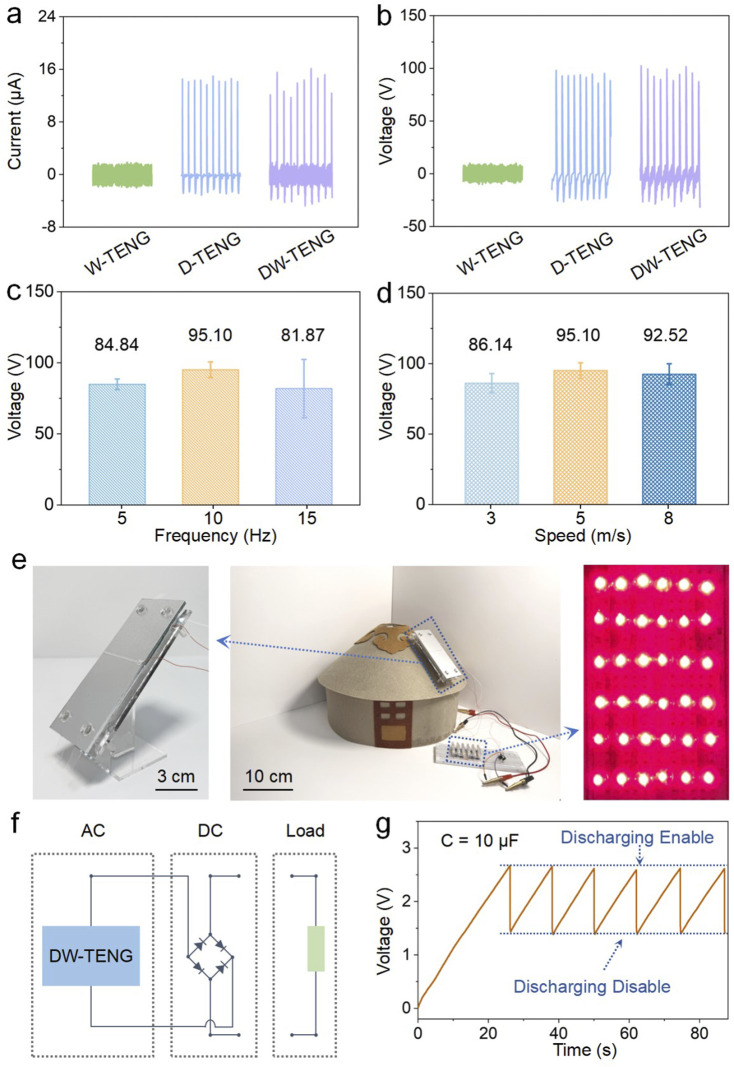
Output performance and application of the DW-TENG. **(A)** Output current and **(B)** voltage of the W-TENG, D-TENG, and DW-TENG (size = 100 × 50 mm, frequency = 10 Hz, wind speed = 5 m/s, height = 30 cm, top electrode length = 50 mm, top electrode width = 2 mm, and angle = 45°). **(C)** Output voltage of the DW-TENG under different droplet frequencies (size = 100 × 50 mm, wind speed = 5 m/s, height = 30 cm, top electrode length = 50 mm, top electrode width = 2 mm, and angle = 45°) and **(D)** different wind speeds (size = 100 × 50 mm, frequency = 10 Hz, height = 30 cm, top electrode length = 50 mm, top electrode width = 2 mm, and angle = 45°). **(E)** Optical photographs of the DW-TENG lit 36 LEDs in a yurt application scenario. **(F)** Circuit diagram of the DW-TENG. **(G)** Voltage history of the capacitor in the charging process. (size = 100 × 50 mm, frequency = 10 Hz, wind speed = 5 m/s, height = 30 cm, top electrode length = 50 mm, top electrode width = 2 mm, angle = 45°, and capacitance = 10 μF).

Considering the actual application scenarios of rain and wind, to make the operating conditions of the DW-TENG closer to the raindrop falling frequency and the annual average wind speed, the droplet falling frequency was selected to be 10 Hz, and the wind speed condition was selected to be 5 m/s. The D-TENG and W-TENG portions of the hybridized device were connected in parallel to carry out the signal test. The results are shown in Figures 4A, B. The output current and voltage were, respectively, 1.40 μA and 7.60 V for blowing wind only, 14.30 μA and 92.71 V for dripping droplet only, and 13.86 μA and 95.10 V for simultaneous action of the droplet and wind. Due to the simultaneous operation, the droplet position was deviated by the wind interference, resulting in a signal that was not as stable as when working alone. In addition, because the current signal peak due to the droplet was generated in a very short period of time of 2 ms, the hybridized current peak was lower than the peak of the droplet current alone on average, although the hybridized signal could also reach 16.12 μA at its highest point. In order to further investigate the power generation performance of the device under different rainfall and wind speeds, different droplet frequencies were selected to simulate light, medium, and heavy rain scenarios, and different wind speeds were selected to simulate light, medium, and strong wind environments. Figure 4C shows the output signals of the hybridized device when the water droplet frequency is 5 Hz, 10 Hz, and 15 Hz, and the output voltage reached 84.84 V, 95.10 V, and 81.87 V, accordingly. Figure 4D shows the output signals of the hybridized device when the wind speed is 3 m/s, 5 m/s, and 8 m/s, and the output voltage reached 86.14 V, 95.10 V, and 92.52 V, correspondingly. Higher peak current or voltage did not occur in the heavy rain and windy scenarios, mainly because the droplet signals were influenced by the blowing wind; the droplet position had some deviation, resulting in unstable signals. The details of the current and voltage signals are given in Supplementary Figure S7. To further explore the possibilities of DW-TENG applications, the device was placed on the roof of a yurt model, as shown in Figure 4E, and the electricity generated from the device (see Supplementary Figure S8A for details of the side of the device) was rectified and connected to the power-using device for wiring, as shown in Figure 4F, which can light up at least 36 LED lights at once. When driving external loads, all three wires of the DW-TENG are connected to the AC terminal of the rectifier bridge, and the DC output of the rectifier bridge is connected to the two electrodes of the LEDs, thus realizing the conversion of the AC power of both the D-TENG and W-TENG into DC power and supplying power to external loads at the same time. Supplementary Movie S1 shows the power generation process of the device under dripping and blowing conditions on a yurt model. As shown in Figure 4G, after charging the capacitor to 2.5 V for the first 26 s, a cluster of LEDs can be lit approximately once every 12 s on average. In the future, DW-TENG arrays (Supplementary Figure S8B) can be placed on rooftops or other spacious platforms in remote areas such as yurts to collect more environmentally dispersed energy for driving power-using devices such as lights and monitoring ambient temperature, humidity, or wind speed, etc.

## 4 Conclusion

In this work, a hybridized nanogenerator, DW-TENG, was proposed, which can simultaneously harvest droplet energy and wind energy. The design of the DW-TENG successfully superimposed the electrical energy harvested by the D-TENG and W-TENG portions, and it achieved an output voltage of 95.10 V, a maximum average power of 18.15 μW, and an energy generation of up to 181.54 μJ within 10 s and can charge a 1-μF capacitor to 43.29 V under a simulated environment of medium rain and a medium breeze. In the yurt model application scenario test, a single DW-TENG device successfully illuminated a cluster of 36 LEDs every 12 s. This work provides a new idea and possibility for online monitoring and IoT system construction in remote areas.

## Data Availability

The raw data supporting the conclusions of this article will be made available by the authors, without undue reservation.
