# Supplementary material for: Hybridized triboelectric nanogenerators for simultaneously scavenging droplet and wind energies
Source: Front Chem. 2025 Jan 10;12:1538660. doi: 10.3389/fchem.2024.1538660 (PMC11780274; doi:10.3389/fchem.2024.1538660)
Supplement: Supplementary file 2 [file DataSheet1.docx]

Hybridized nanogenerator for simultaneously
scavenging droplet and wind energies

Chaosheng Hu^1,3†^, Chengmin Bao^2†,^*, Yang Liu^2^, Yingzhan Yan^1,3^, Yanan Bai^4^, Qian Xu^1,3,^*

^1^Information Science Academy, China Electronics Technology Group Corporation, Beijing 100042, P.R. China.

^2^School of Chemistry and Environmental Engineering, Hohhot Minzu College, Hohhot 010051, P.R. China

^3^National Key Laboratory of Integrated Circuits and Microsystems, Beijing 100042, P.R. China., Country

^4^School of Physical Science and Technology, Guangxi University, Nanning 530004, P.R. China

**† Co-first authors**

*** Correspondence:**

Qian Xu, Information Science Academy, China Electronics Technology Group Corporation, Beijing 100042, P.R. China.

Email: [xuqian199004@163.com](mailto:xuqian199004@163.com)

Chengmin Bao, School of Chemistry and Environmental Engineering, Hohhot Minzu College, Hohhot 010051, P.R. China
Email: hohhotbaochengmin@126.com


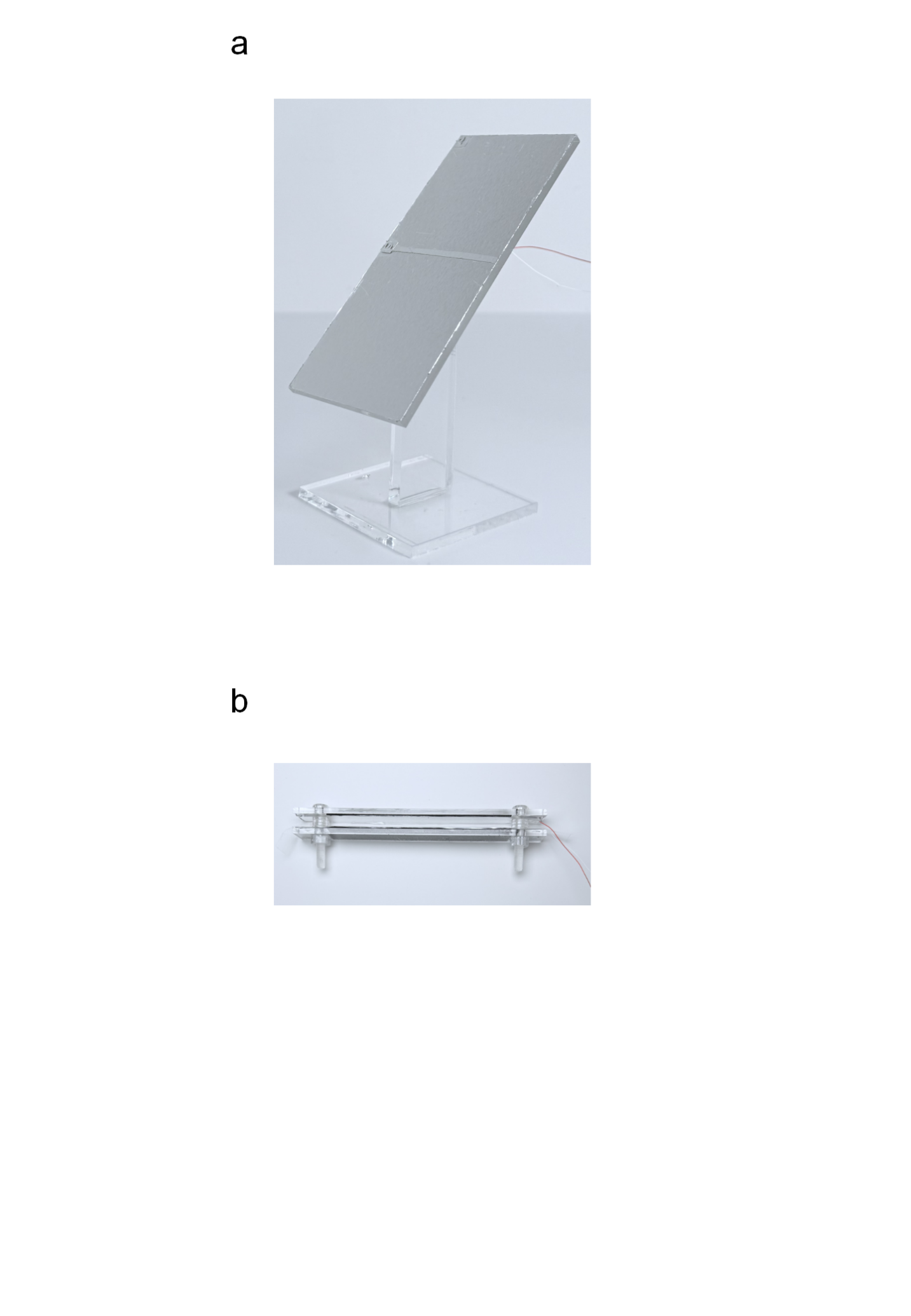


**FIGURE S1 Optical photographs of (a) the D-TENG and (b) the W-TENG.**


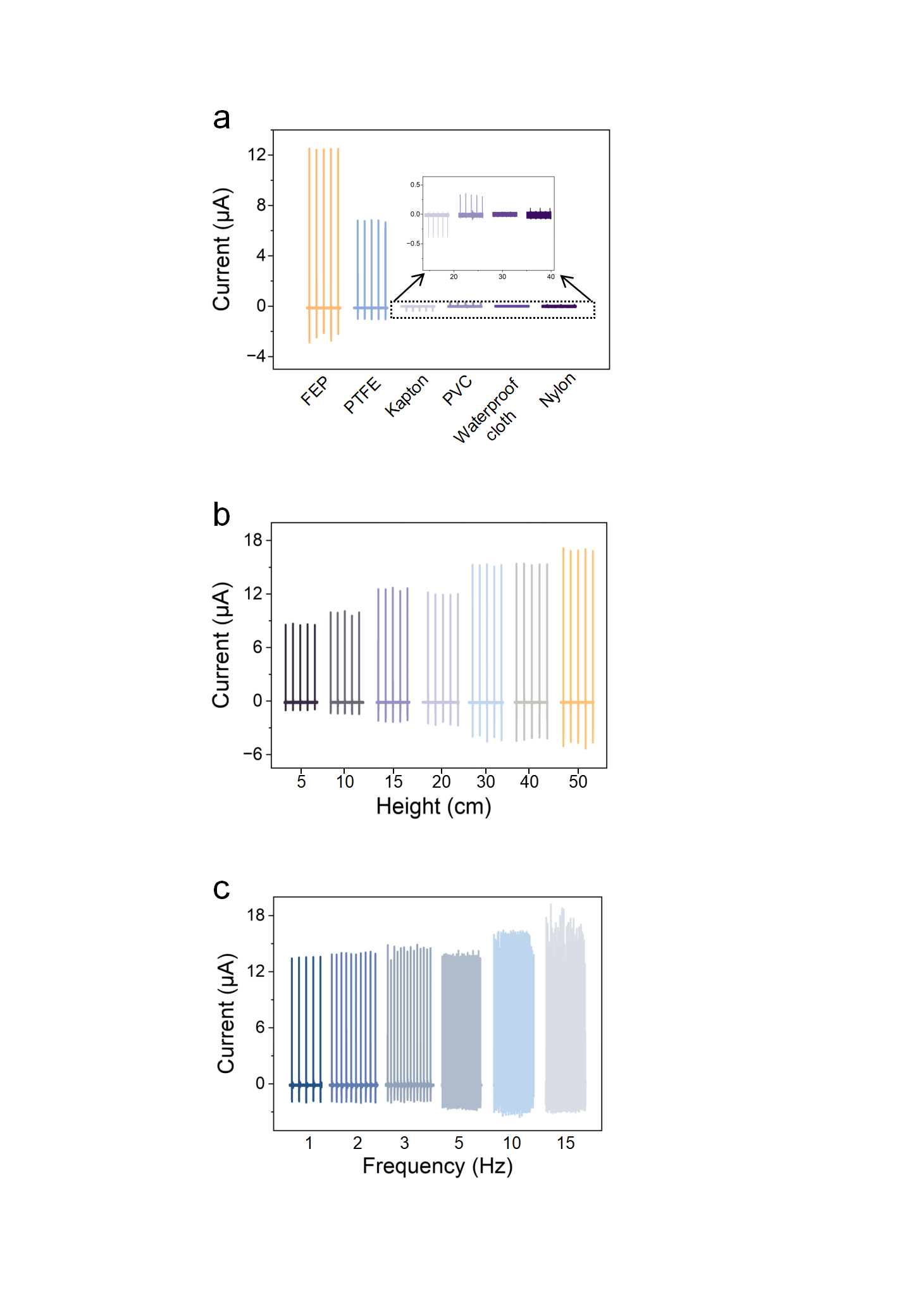


**FIGURE S2 Output optimization of the D-TENG.**

(a) Output current of the D-TENG under different surface materials (size = 30 × 30 mm, frequency = 1 Hz, height = 15 cm, top electrode length = 15 mm, top electrode width = 2 mm, Angle = 45°), (b) different height (size = 100 × 50 mm, top electrode length = 50 mm, top electrode width = 2 mm, frequency = 1 Hz, Angle = 45°), and (c) different droplet frequency (size =100 × 50 mm, height = 15 cm, top electrode length = 50 mm, top electrode width = 2 mm, Angle =45°).


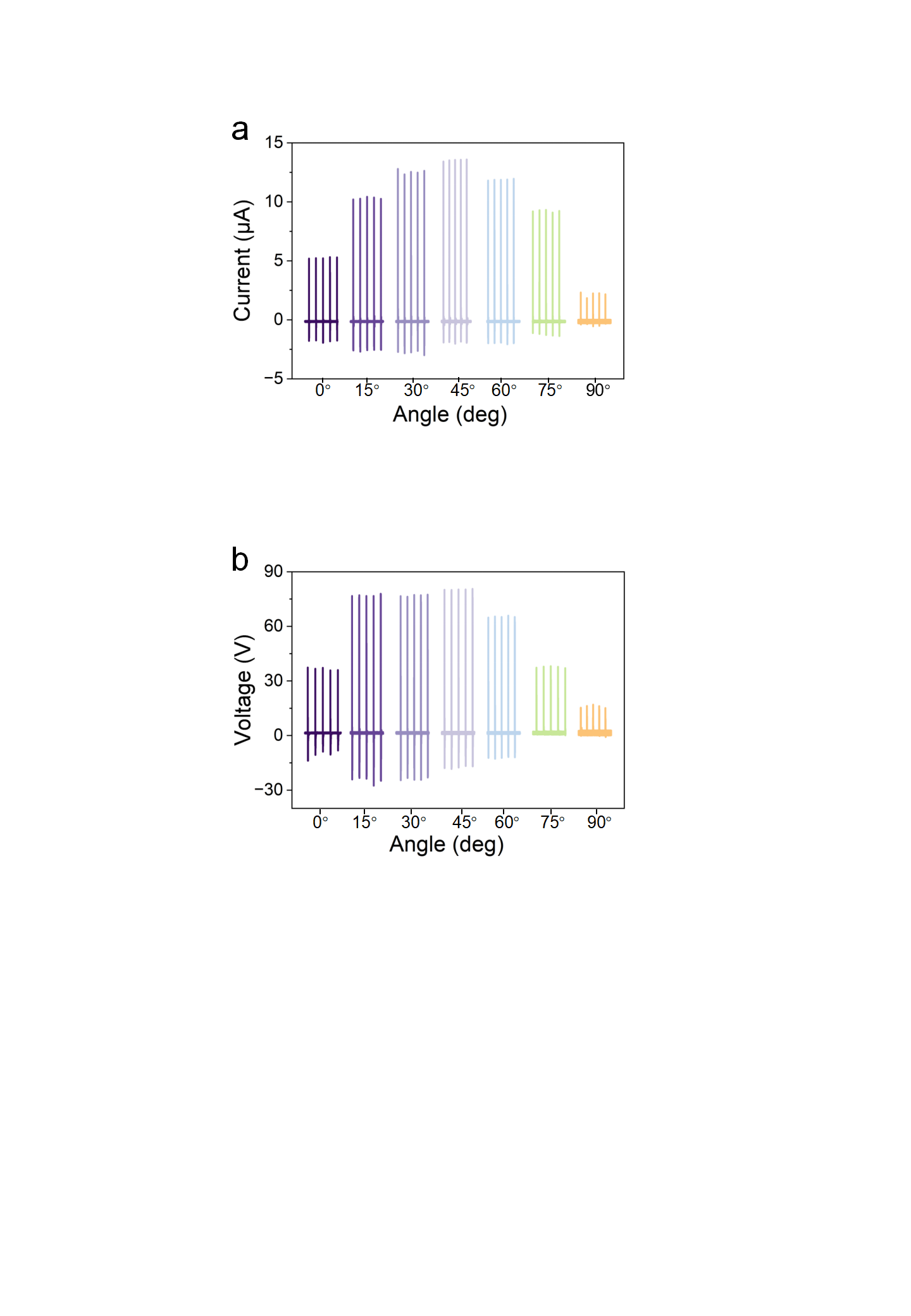


**FIGURE S3 Output (a) current and (b) voltage of the D-TENG under different inclined angle.** (size = 100 × 50 mm, height = 15 cm, frequency = 1 Hz, material = FEP, top electrode length = 50 mm, top electrode width = 2 mm)


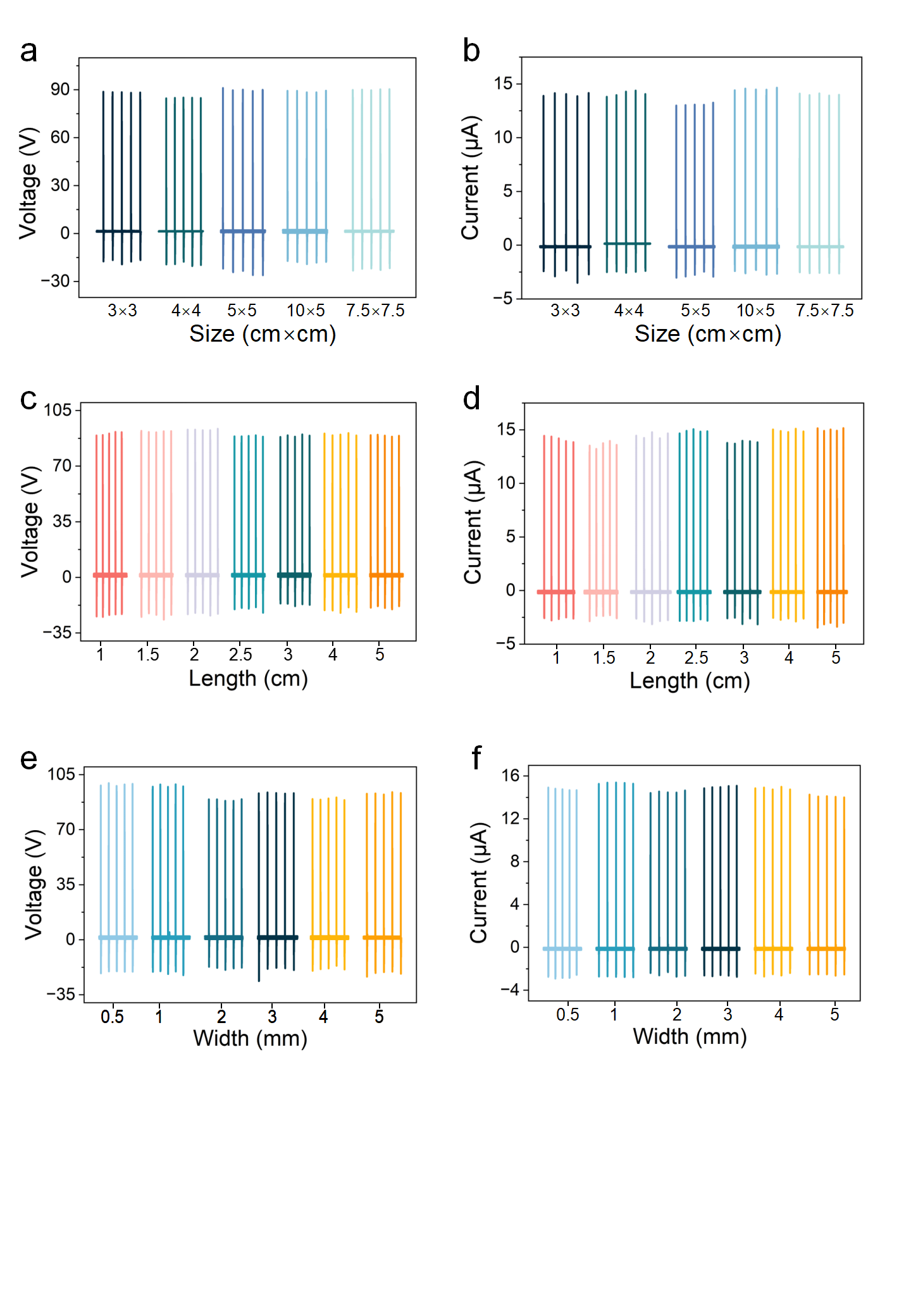


**FIGURE S4** **Output performance of D-TENG under different sizes, lengths and widths of top electrode.** (a) Output voltage and (b) current of the D-TENG of different sizes (frequency = 1 Hz, height = 15 cm, material = FEP, top electrode length = 15 mm, top electrode width = 2 mm, Angle =45°). (c) Output voltage and (d) current of different top electrode lengths (size =100 × 50 mm, height = 15 cm, material = FEP, frequency = 1 Hz, top electrode width = 2 mm, Angle = 45°). (e) Output voltage and (f) current of different top electrode widths (size =100 × 50 mm, height = 15 cm, material = FEP, frequency = 1 Hz, top electrode length = 15 mm, Angle = 45°) of the D-TENG device.


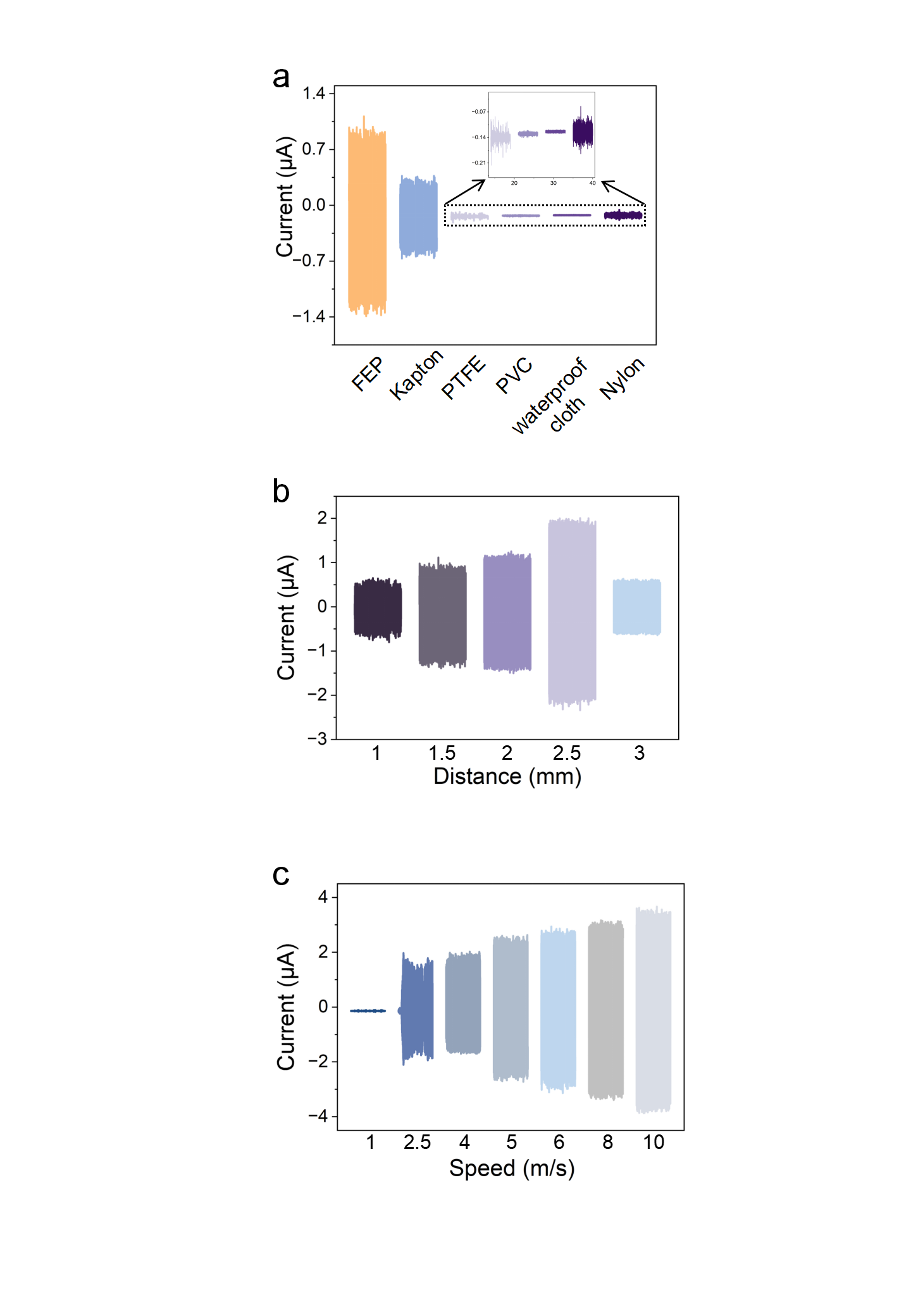


**FIGURE S5 Output optimization of W-TENG.**

(a) Output current of the W-TENG under different surface materials (size =1×10, wind speed =5m/s, distance =1.5mm, Angle =0°), (b) different gap distances between the FEP and Al electrodes (size = 100 × 10 mm, wind speed = 5 m/s, material = FEP, Angle = 0°), and (c) different wind speed (size = 100 × 10 mm, distance = 2.5 mm, Material = FEP, Angle = 0°).


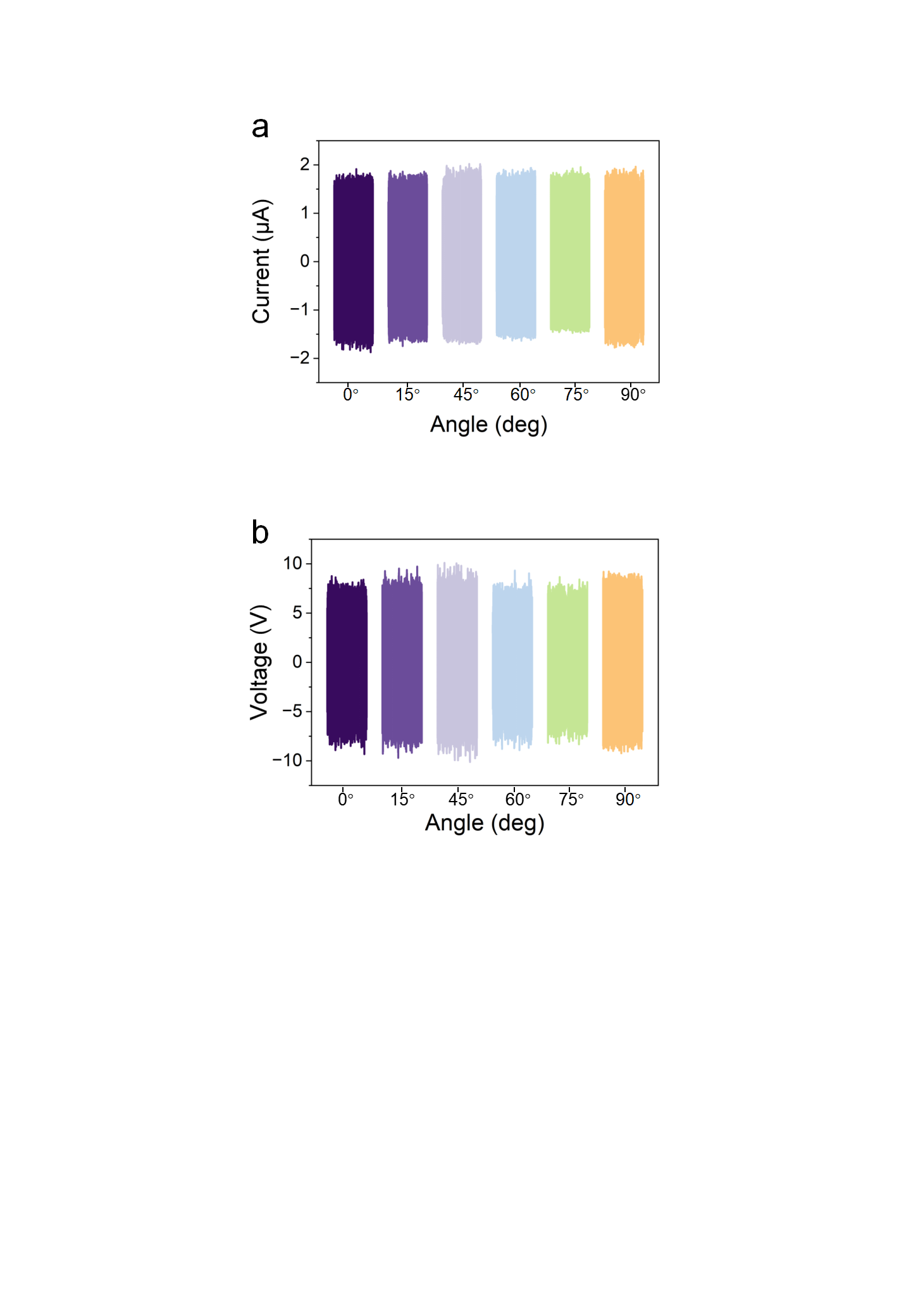


**FIGURE S6 Output (a) current and (b) voltage of the W-TENG under different inclined angle.** (size =100 × 10 mm, wind speed = 5 m/s, distance = 2.5 mm, material = FEP)


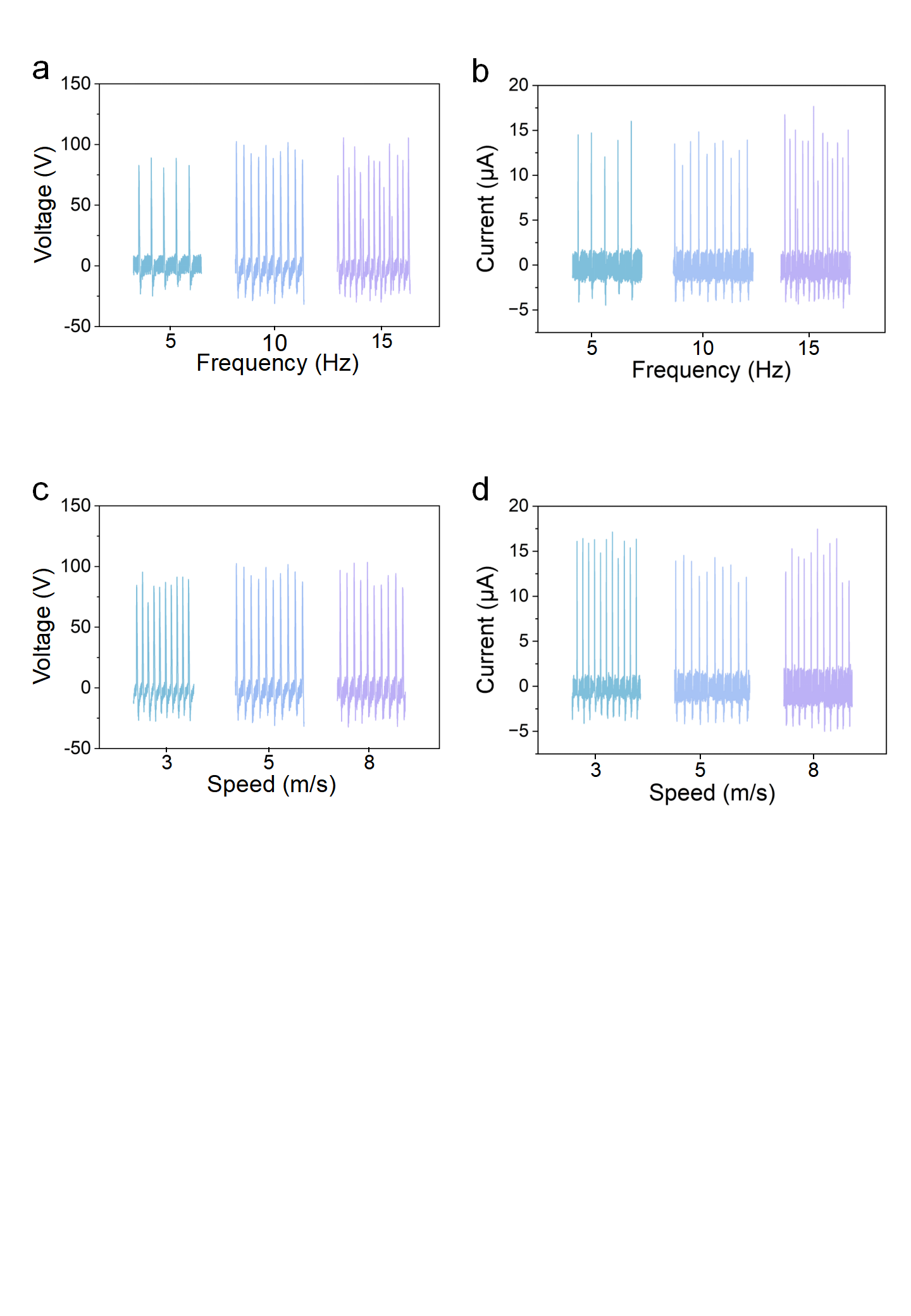


**FIGURE S7 Output optimization of DW-TENG.**

(a) Output voltage and (b) current of the DW-TENG under different droplet frequency (size = 100 × 50 mm, wind speed = 5 m/s, height = 30 cm, top electrode length =50 mm, top electrode width 2mm, Angle =45°). (c) Output voltage and (d) current of the DW-TENG under different wind speed (size =100 × 50 mm, frequency = 10 Hz, height = 30 cm, top electrode length = 50 mm, top electrode width = 2 mm, Angle = 45°).


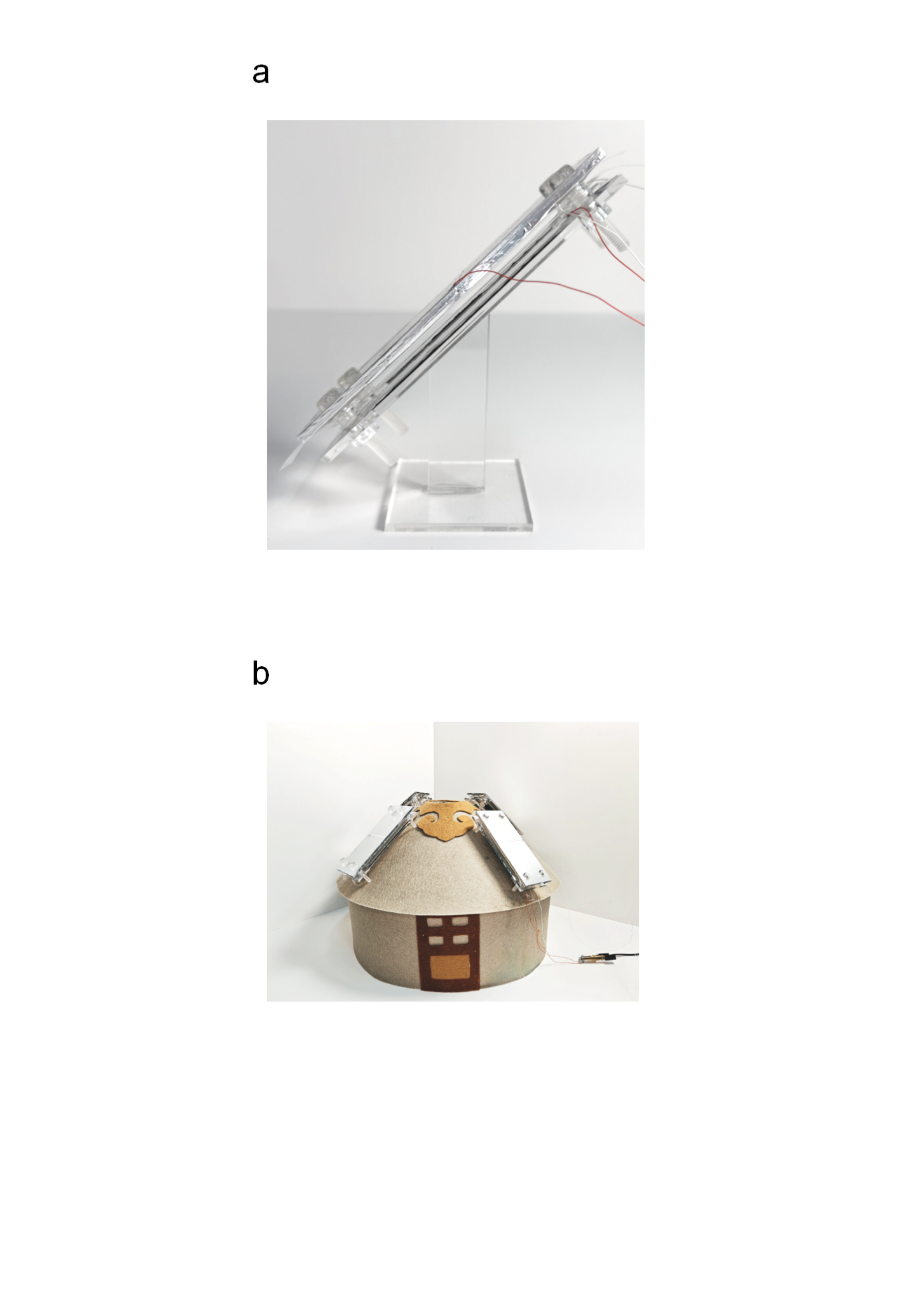


**FIGURE S8** **Optical photographs of (a) the DW-TENG and (b) application scenario.**
